# Fabrication Scaffold with High Dimensional Control for Spheroids with Undifferentiated iPS Cell Properties

**DOI:** 10.3390/cells12020278

**Published:** 2023-01-11

**Authors:** Hidetaka Togo, Kento Terada, Akira Ujitsugu, Yudai Hirose, Hiroki Takeuchi, Masanobu Kusunoki

**Affiliations:** 1 Graduate School of Biology-Oriented-Science and Technology, Kindai University, 930 Nishimitani, Kinokawa 649-6493, Wakayama, Japan; 2 Department of Obstetrics and Gynecology, Graduate School of Medicine, Mie University, 2-174 Edobashi, Tsu 514-8507, Mie, Japan; 3 Faculty of Biology-Oriented-Science and Technology, Kindai University, 930 Nishimitani, Kinokawa 649-6493, Wakayama, Japan

**Keywords:** spheroid, organoid, uniform dimension, scaffold, iPS, undifferentiated nature

## Abstract

Spheroids are expected to aid the establishment of an in vitro-based cell culture system that can realistically reproduce cellular dynamics in vivo. We developed a fluoropolymer scaffold with an extracellular matrix (ECM) dot array and confirmed the possibility of mass-producing spheroids with uniform dimensions. Controlling the quality of ECM dots is important as it ensures spheroid uniformity, but issues such as pattern deviation and ECM drying persist in the conventional microstamping method. In this study, these problems were overcome via ECM dot printing using a resin mask with dot-patterned holes. For dot diameters of φ 300 μm, 400 μm, and 600 μm, the average spheroid diameters of human iPS cells (hiPSCs) were φ 260.8 μm, 292.4 μm, and 330.7 μm, respectively. The standard deviation when each average was normalized to 100 was 14.1%. A high throughput of 89.9% for colony formation rate to the number of dots and 89.3% for spheroid collection rate was achieved. The cells proliferated on ECM dots, and the colonies could be naturally detached from the scaffold without the use of enzymes, so there was almost no stimulation of the cells. Thus, the undifferentiated nature of hiPSCs was maintained until day 4. Therefore, this method is expected to be useful in drug discovery and regenerative medicine.

## 1. Introduction

In drug development, in vitro tests and cell cultures are essential for selecting candidate substances, while in vivo tests and animal experiments are essential for confirming drug efficacy and toxicity. In addition, chemical legislation (REACH, CLP, Cosmetics Directive, etc.) requires animal testing, because standard in vitro tests have low specificity, and reports have shown that non-genotoxic chemicals often falsely show genotoxicity in vivo [1,2,3]. However, animal experiments using chemicals have come under scrutiny in recent decades, and a paradigm shift is underway in several countries to prohibit animal experimentation, considering the perspective of animal welfare. Consequently, the European Union has banned the use of laboratory animals in in vivo follow-up studies of cosmetic ingredients since 2009 [4]. However, animal experimentation remains essential in drug discovery and medical device development. Nevertheless, the demand for an alternative technology that can mimic tissue dynamics through cell culture is expected to increase in the future. Cell culture is an important technique in basic and preclinical research and is a cost-effective way to reduce the number of experimental animals in drug discovery.

Previously, cancer cells have been studied to verify the efficacy of therapeutic drugs [5], and the three-dimensional (3D) structures of bones and cartilages have been cultured to reproduce mechanical properties [6,7]. Current culture methods are expected to used pluripotent stem cells that differentiate into target tissues for application in drug discovery and regenerative medicine, such as embryonic stem cells and induced pluripotent stem cells (iPSCs). In recent years, spheroids, or cell aggregates, as well as mini-organs named organoids, have been used in studies to mimic various organs, such as the liver [8,9], kidneys [10,11,12], and intestine [13,14,15]. A 3D structure is necessary to express multicellular functionality. Therefore, there is a high demand for stable methods for preparing spheroid and organoids, and various 3D culture methods have been proposed. Conventional 3D culture methods can be broadly classified into four types [16]: micropattern forced flotation [17,18], hanging drop [19,20,21], swirl culture or spinner flask [22,23,24,25,26,27,28,29,30], and force-driven methods [31,32,33]. The micro-patterned forced suspension method uses low-adhesion well plates to enable cells precipitated in the microwells to adhere to each other in a 3D manner. In the hanging drop method, the surface tension of the culture medium is used to form droplets, and the interface with the air is used as a microwell. The spinner flask or swirling culture (bioreactor) is a method in which a culture medium containing dispersed cells is continuously swirled and agitated so that the cells collide to form spheroids. Force-driven methods, such as magnetic force, electric field, and acoustic tweezers are known. Other methods have also been developed in recent years. For example, 3D printing can artificially create 3D shapes using a computer-controlled arrangement of heterogeneous cells with a gel-like culture medium as the filler. These existing methods exhibit excellent features, such as spheroid dimension control, reduced damage and stimulation to cells, simple procedures, a high success rate, mass production, high throughput, and low cost, but none of them satisfy all these requirements simultaneously.

In a previous study [34], we proposed a simple and hypoallergenic method that can simultaneously satisfy all the requirements of the existing methods. The conventional methods often focus on preventing cell adhesion to the scaffold. However, we considered it important to spheroidize the cells while maintaining various extracellular matrices for adhesion to the scaffold [35]. When cells are seeded on a fluoropolymer-coated substrate with extracellular matrix (ECM) dots, they form monolayer colonies only on the ECM, and these spontaneously exfoliate and agglutinate into spheroids by themselves under a weak trigger, such as pipetting. This method, which consists of the “cell culture” and “spheroidized” stages, has several features, as described below. Spheroids with uniform dimensions can be obtained using ECM dot arrays of a certain size. Colonies consisting of freshly grown cells on ECM dots are detached without using enzymes, so the probability of degraded cells entering the spheroids is extremely low. The ECM does not bind strongly to this scaffold due to the chemically stable nature of the fluoropolymer. Since our method uses the very weak force with which this ECM adheres to the substrate, it is not necessary to consider the effects of polarity, as is seen with conventional scaffold materials. Therefore, one of the features of our method is that the ECM used is not limited. Except for the use of this scaffold, the method utilizes a normal cell culture, and spheroids can be obtained easily without special techniques. In addition, spheroids larger than 500 µm could be produced using an established cell line, and the cells that make up the spheroids showed a survival rate of nearly 90%. Thus, the method satisfies the criteria of high success rates, mass production, high efficiency, and low cost all at the same time. We believe that these characteristics are advantageous for inducing stem cell differentiation. This is because when pluripotent stem cells differentiate into cells of various tissues, the first half of the process is induced using cytokines and low molecular weight compounds in two-dimensional (2D) culture; in the second half, induction is carried out by forming spheroids and organoids in 3D culture [36,37,38].

In our previous experiments [34], ECM dots were produced using the microstamping method to demonstrate its feasibility and effectiveness in principle. Although the microstamping method is a simple technique, it is highly dependent on the skill of the experimenter. Microstamped ECM patterns sometimes exhibit defects, such as blurring, dimensional deviations, and variations in the amount of ECM. These variations are not easy to prevent; therefore, more versatile alternatives are required. This spheroid fabrication method shows clear effectiveness, and its practicality would be increased if a uniform ECM pattern could be easily produced. This was the primary purpose of this study. Specifically, we investigated a method to create uniform ECM dot arrays using negatively patterned resin masks. The second purpose was to experimentally show that the new method can be applied to stem cells, as it had only been tested with established cell lines in a previous study [34]. The undifferentiated nature and their ability to differentiation of the cells in the colony state just prior to forming spheroids were confirmed.

## 2. Materials and Methods

### 2.1. Spheroid Culture Scaffold Fabrication

A fluoropolymer surface was formed by coating a glass substrate with CYTOP™ (CTL-107MK; AGC Chemicals, Tokyo, Japan). This resin exhibits fluoropolymer properties, such as water repellency, oil repellency, and chemical resistance, as well as high transparency. It can also be used for cell culture observation using an inverted microscope, as it is not fluorescent. The material was immersed in a dip solution containing CYTOP™ and a thinner solution (CT-Solv.100; AGC Chemicals) to coat the glass substrate. Pull-out and curing conditions were as recommended by the manufacturer.

### 2.2. Resin Mask Fabrication for ECM Patterning

**Figure 1:**
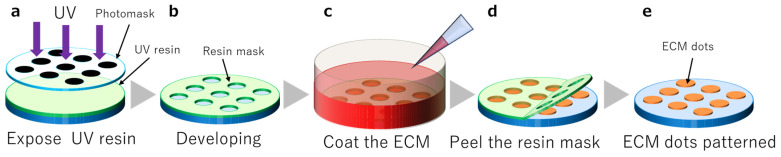
Resin mask fabrication and extracellular matrix (ECM) coating. (**a**): UV resin exposure, (**b**): Dot pattern development, (**c**): ECM coating, (**d**): Resin mask peeling, (**e**): Completion of ECM dots.

A UV-curable resin (1209-M-UR-SC; Dymax, Torrington, CT, USA) was used as a resin mask to pattern the ECM dots. It was cured using UV light at 385 nm or 405 nm. An i-line mask aligner was used to expose the resin. Photomasks with 300 µm, 400 µm, 600 µm, and 800 µm diameter dots were used to create a UV shadow (Figure 1a,b).

### 2.3. ECM Patterning

ECM substrates, including iMatrix-511 silk (Nippi, Tokyo, Japan), were diluted in Dulbecco’s modified Eagle’s medium (DMEM; Nacalai tesque, Kyoto, Japan). The substrate was placed in a dish with the opening of the resin mask facing upwards. The ECM substrate was poured and incubated at 37 °C and 5% CO_2_ for 24 h (Figure 1c). The ECM was allowed to settle and coat the substrate surface while resting with the aperture facing up. After incubation, the resin mask was peeled (Figure 1d), and the substrates were washed with phosphate-buffered saline (PBS) (-) (Figure 1e).

### 2.4. Human iPSC (hiPSC) Culture

HiPSCs (253G1 and 409B2) were obtained from the RIKEN BioResource Research Center (RIKEN BRC, Tsukuba, Ibaraki, Japan), Japan. The hiPSCs were maintained via the following method: 10% fetal bovine serum (FBS) (BioWest, Nuaillé, Maine-et-Loire, France)/DMEM (Nacalai tesque) containing 0.42 µL/cm^2^ iMatrix was placed in a culture dish and incubated at 37 °C, 5% CO_2_, 95% air for 24 h. For passage, the medium was aspirated, washed with PBS(−), and incubated with TrypLE Select (1×), no phenol red (ThermoFisher scientific, Waltham, MA, USA), for 3–5 min at 37 °C. The cells were then collected by adding 10% FBS/DMEM and centrifuged at 1500 rpm for 3–5 min at room temperature, and the supernatant was aspirated. The cells were then suspended in StemFit (AK02N; Ajinomoto, Tokyo, Japan) containing 10 µM Y27632 (Nacalai tesque) and a 100-fold diluted penicillin-streptomycin mixed solution (Nacalai tesque) and seeded at 1:3. The next day, as well as every 1–2 days thereafter, the medium was replaced with StemFit until passaging. Passages were made every 3–4 days.

### 2.5. Spheroid Preparation

**Figure 2:**
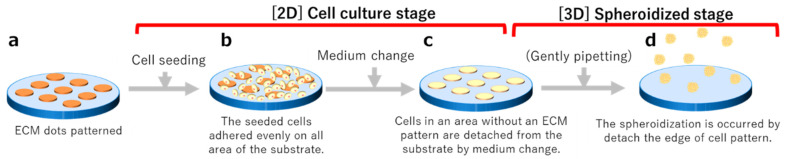
Schematic diagram of the spheroid production process. (**a**) Matrix transferred via stamping. (**b**) Cell adhesion occurs one day after seeding. (**c**) Cell colony formation a few days after seeding. (**d**) After cell detachment, spheroids form in suspension.

Cells were evenly seeded onto the fluoropolymer-coated substrate with ECM dots (Figure 2a) prepared as described in Section 2.3 (seeding concentration: 1 × 10^5^/cm^2^ to measure spheroid size, 5 × 10^3^/cm^2^ to 5 × 10^4^/cm^2^ to confirm undifferentiation) (Figure 2b). After a few days of culture (described in Section 2.4), the cells proliferated and formed colonies only on the ECM-coated area (Figure 2c). The cell colonies were detached through gentle pipetting, followed by culturing in suspension for a few days until spheroid formation (Figure 2d). All cultures were performed on fluoropolymer-coated glass substrates in a 5-well Petri dish (Naka Medical, Tachikawa, Tokyo, Japan).

### 2.6. Evaluation of Cell Patterning

The dimensions of the ECM dots, 2D colonies, and spheroids were observed using phase-contrast microscopy, and they were then photographed and dimensioned using an IX71N-22FL/PH (Evident, Tokyo, Japan). Although the silhouettes of the cell colonies and spheroids were almost circular, their diameters were defined as the average of the vertical and horizontal diameters.

### 2.7. Measurement of Spheroid Viability Using Trypan Blue Staining

The cells of the 2D culture and spheroids were dissociated using trypsin/EDTA(Nacalai Tesque) buffer, and a single cell suspension was obtained. The cells in the suspension were then stained with trypan blue (Nacalai Tesque) to determine the number of viable cells.

### 2.8. Differentiation of iPSCs on Substrates

Confluent hiPSC colonies on substrates were differentiated into early endoderm, mesoderm, and ectoderm. Those differentiated into early endoderm were cultured in RPMI1640 (Nacalai Tesque) containing 2 mM L-alanyl-glutamine (Nacalai Tesque) and 1 × B27 supplement without insulin (Thermo Fisher Scientific, Waltham, MA, USA), 100 ng/µL recombinant Activin A (AproScience, Tokushima, Japan), 3 μM CHIR99021 (Nacalai Tesque), and 100 nM wortmannin (Cayman Chemical, Ann Arbor, MI, USA) for 2 days. The wortmannin was then excluded from the above medium for another 2 days. For early differentiation into mesoderm, cells were grown for 3 days in RPMI 1640 medium containing 2 mM L-alanyl-glutamine and 1 × NS supplement (FUJIFILM Wako Pure Chemical, Osaka, Japan) with 10 ng/μL recombinant Activin A and 30 ng/μL recombinant human BMP4 (Peprotech, Cranbuury, NJ, USA). Next, 3 μM CHIR99021 and 100 nM wortmannin were added to the medium and cultured for 3 days. For early differentiation into ectoderm, the StemXVivo Ectoderm kit (R & D systems, Minneapolis, MN, USA) was used and manufacture’s protocol were followed. MA, USA), mouse monoclonal anti-TRA-1-81 (1:1000; 4745, CST), mouse monoclonal anti-SSEA4 (1:1000; 4755, CST), rabbit anti-NANOG (1:500; 4903, CST), rabbit monoclonal anti-FOXA2/HNF3β (1:400; 8186S, CST), and mouse monoclonal anti-SOX17 (1:100; TA500044, Origene, Rockville, MD, USA) as early endoderm markers, rabbit polyclonal anti-Cardiac Troponin T (1:100; 15513-1-AP, Proteintech, Rosemont, IL, USA) and mouse monoclonal anti-PAX2 (1:100; MBS4380650, MyBioSourse, San Diego, CA, USA) as early ectoderm markers, and goat polyclonal anti-OTX2 (1:100; SC031B, R & D systems) and rabbit monoclonal anti-β Tubulin (1:100; 80713-1-RR, Proteintech) as early ectoderm markers. The cells were washed three times in PBS(−) and incubated with Alexa Fluor 488- or 594-conjugated Donkey anti-rabbit or anti-mouse IgG (1:1000; ThermoFisher scientific) for 1 h at room temperature. After a few drops of VECTASHIELD (H-100, Vector Laboratories, Newark, CA, USA) were applied, an upright microscope (BX43, Evident) modified to fluorescent specifications, a dedicated camera (DP-74, Evident), and cellSens standard imaging software (Evident) were used to acquire images.

## 3. Results

### 3.1. Dimensional Reproducibility of the Resin Mask

**Figure 3:**
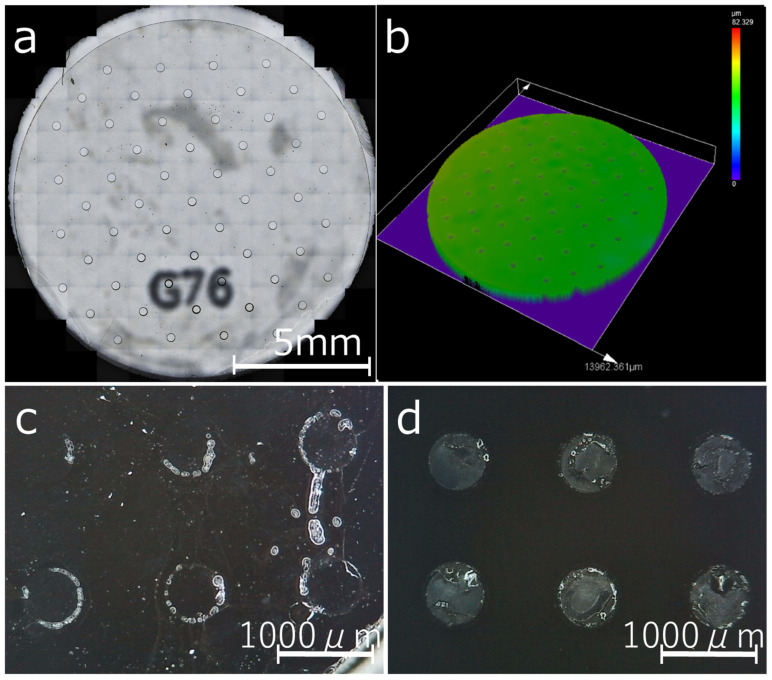
Observation of the top surface of a resin mask using a laser microscope. (**a**) Optical imaging of the substrate and resin mask surface. (**b**) Three-dimensional image of a resin mask surface. Matrigel patterns produced using microstamping and a resin mask (drying for pattern visualization). (**c**) Pattern formation using microstamping. (**d**) Pattern formation using a resin mask.

First, we evaluated the uniformity of the hole diameter of the UV-cured resin mask using a 300 μm dot diameter glass mask. One substrate had 57 holes arranged in an array (Figure 3a). A total of 171 holes were measured for the three substrates, and mean values and standard deviations were obtained. The hole diameter of the resin mask was 328.3 ± 3.8 μm. This demonstrated the possibility of creating masks with sufficiently uniform hole diameters. Next, ECM (Matrigel) dots were fabricated using a resin mask and compared with those of the conventional microstamp. The fluoropolymer and ECM were difficult to observe in the liquid, as they were extremely thin and transparent. Therefore, we intentionally dried them and observed them using a microscope to improve visibility. Figure 3c shows an example of a failed ECM dot prepared via φ 600 µm microstamping, exhibiting blurring, chipping, bleeding, and dirt on the base surface outside the dot. In contrast, Figure 3d shows the ECM dot pattern obtained using the resin mask, where a uniform pattern could be obtained without failure, no matter how many times it was repeated.

### 3.2. Culturing hiPSCs on the iMatrix Pattern and Confirming Viability

**Figure 4:**
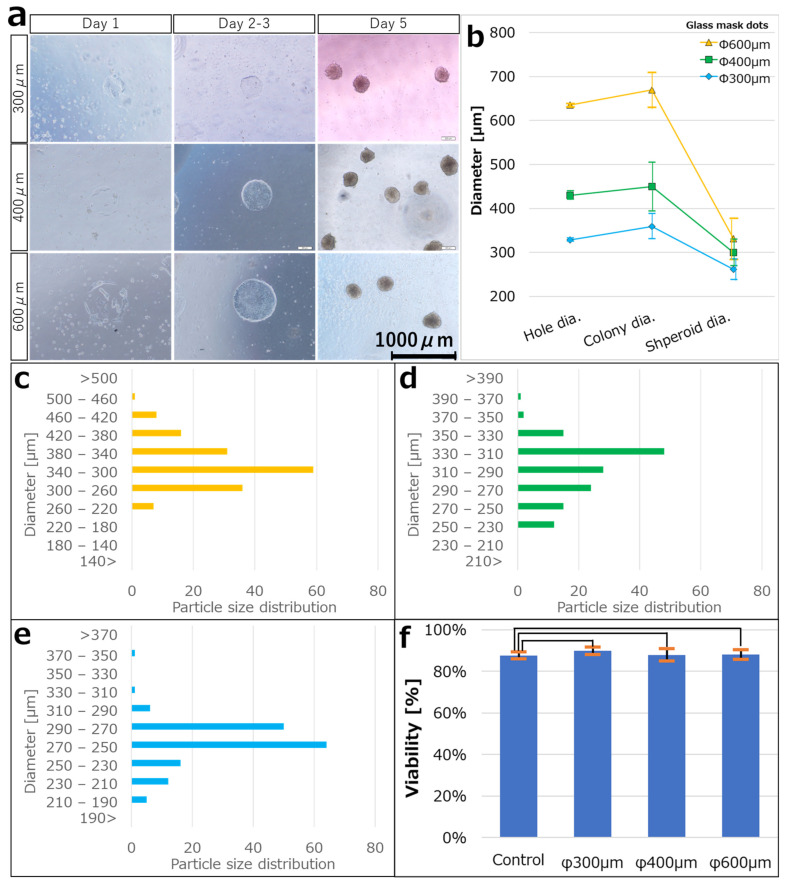
(**a**) Bright-field images for each day after seeding hiPSCs on iMatrix-coated substrates of each dot size (300 μm, 400 μm, and 600 μm). The hiPSCs adhered to the substrate on day 1 and proliferated into colonies of the same size as the iMatrix pattern on days 2–3, and the colonies dissociated from the substrate on days 3–4. The colonies were then incubated in a three-dimensional suspended state, and spheroids were formed on day 5. (**b**) The average diameter of the dot patterns during each step. Error bars indicate the standard deviation. Particle size distribution of spheroids produced at patterns of (**c**) 300 μm (N = 155), (**d**) 400 μm (N = 145), and (**e**) 600 μm (N = 158). (**f**) Viability confirmation by trypan blue staining. Student t-test was performed to compare survival rates between the 2D colonies and the spheroids (*p* = 0.05). *p* < 0.05 was considered statistically significant. Data are expressed as means ± standard deviations. The relationships were not statistically significant in all of these cases.

HiPSCs were seeded on the ECM-patterned substrate, slight cell adhesion was observed on each ECM dot on day 1, and cells outside the ECM dots were detached. The medium was gently changed every 2–3 days to prevent the cells from detaching, and the cells formed 2D circular colonies based on the ECM pattern after a few days, with an average colony diameter of 359.3 ± 28.4 µm at N = 155. The colonies were detached from the substrate through weak pipetting, cultured in suspension, and spontaneously formed spheroids after a few more days. The average spheroid size was 261.3 ± 23.5 µm at N = 155 (Figure 4a).

Similarly, ECM-patterned substrates were fabricated using a glass mask with φ 400 μm and 600 μm diameter dots. Their respective hole diameters were 429.9 ± 9.6 μm (N = 171) and 634.8 ± 3.5 μm (N = 171). After the hiPSCs were seeded on the ECM-patterned substrate, spheroids were obtained using the same process as in the case of 300 μm diameter dots. The average colony diameters were 449.6 ± 55.6 μm (N = 146) at 400 μm and 669.6 ± 39.8 μm (N = 160) at 600 μm. The average spheroid sizes were 299.9 ± 30.2 μm (N = 145) and 330.7 ± 46.75 μm (N = 158), respectively. The colony production efficiency was 89.9% and the spheroid collection rate was 89.3% relative to the number of resin mask patterns fabricated on the substrate (Figure 4a–e). In addition, trypan blue staining was performed to determine the viability of the spheroids collected from the colonies. The survival rate of the collected spheroids in trypan blue was 88.13 ± 2.26% (N = 5) in 2D culture (the control), 87.65 ± 1.62% (N = 5) at 300 μm, 89.82 ± 1.80% (N = 5) at 400 μm, and 87.88 ± 2.94% (N = 8) at 600 μm (Figure 4f).

### 3.3. Confirming Undifferentiation in hiPSCs Seeded on iMatrix-Coated Substrates

**Figure 5:**
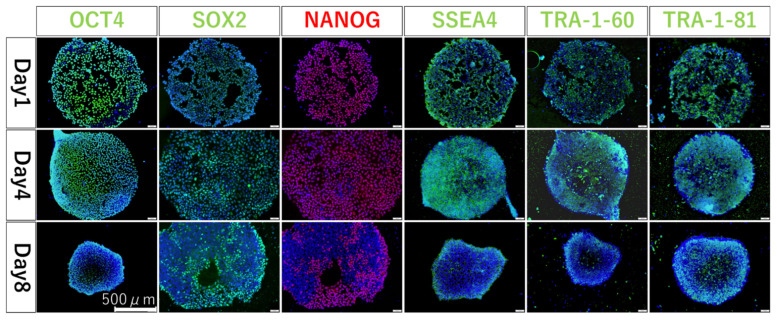
Fluorescent immunostaining results at 1, 4, and 8 days after seeding. Blue coloration shows where cell localize and emit green (OCT4, SOX2, SSEA4, TRA1-60, and TRA-1-81) or red (NANOG) depending on the localization of each protein.

To investigate whether the undifferentiated ability was maintained when hiPSCs were seeded and cultured on fluoropolymer-coated substrates, the localization of undifferentiated marker proteins was examined. hiPSCs were seeded at 5 × 10^4^/cm^2^ and 5 × 10^3^/cm^2^ and were nearly confluent on the following day and on day 4, and immunostaining was performed. The localization of OCT3/4, SOX2, NONOG, SSEA4, TRA-1-60, and TRA-1-81 proteins was confirmed in all hiPSCs on both days. In addition, hiPSCs were seeded at 5 × 10^3^/cm^2^ and cultured to over confluent state on day 8, and immunostaining was performed. Protein localization was lower than that on day 4 in all hiPSCs (Figure 5).

### 3.4. Confirming Differentiation in hiPSCs Seeded on iMatrix-Coated Substrates

**Figure 6:**
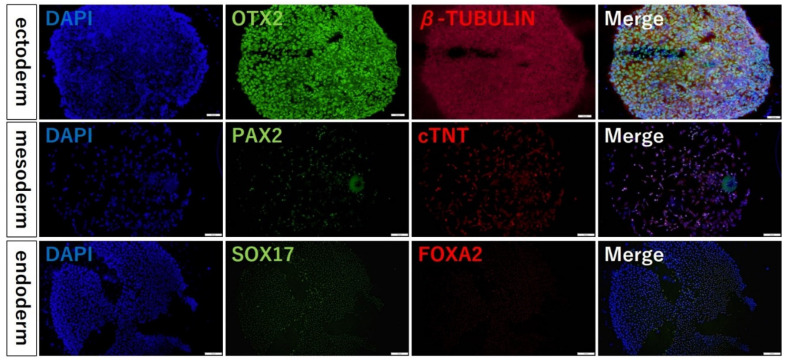
Fluorescent immunostaining results of the multidifferentiation abilities. Blue coloration shows where the cells localize, and they emit green or red (FOXA2, SOX17, PAX2, cardiac Troponin T, OTX2, OCT4, β tubulin) depending on the localization of each protein.

To confirm the multidifferentiation ability of the colonies of iPSCs formed on the fluoroplastic-coated substrates, we differentiated them into endoderm, mesoderm, and ectoderm and checked protein localization. The localization of the early endoderm markers, FOXA2 and SOX17, the early mesoderm markers, PAX2 and Cardiac Troponin T, and the early ectoderm markers, OTX2 and β tubulin, were confirmed (Figure 6).

## 4. Discussion

The scaffold in this study was made possible by the combination of the fluoropolymer and the ECM, which is strong enough to hold cells and weak enough to allow colonies to detach spontaneously. Fluoropolymers are chemically inert because they are nonpolar and have strong C-F bonds. Therefore, we selected this coating material as the substrate as it is bioinert and can be used as a safe and non-toxic material in vivo. In general, fluoropolymers are difficult to adhere to, and for this reason, many studies [39,40,41,42,43,44,45,46,47,48,49,50] have attempted to improve adhesion through surface modification. In contrast, the spheroid fabrication method that we developed uses van der Waals forces that work even on such chemically stable fluoropolymers. We believe that the resin masks fabricated in this study were fixed to the substrate via the same mechanism as that of ECM adhesion. The van der Waals force is strongly affected in the vertical direction, depending on the surface area. However, the resin mask could be easily peeled off from the edge. The cell culture results suggested that no component from the resin mask remained on the fluoropolymer surface after it was peeled off. The weak adhesion of the resin mask to the fluoropolymer is very useful because it creates a temporary mask for the ECM coating that can be easily peeled off.

One of the features of the proposed method is that spheroid diameter variability can be kept extremely low. However, this is principally governed by the uniformity of the ECM dot diameter. The cells that settle outside the ECM dots during seeding are washed away during the medium exchange. The cells adhering to the ECM dots are inhibited from growing outward on the surface where the fluoropolymer is exposed. Therefore, the size of the ECM dots determines the size of the colonies that are obtained. The spheroid size is determined by the number of aggregating cells. Thus, controlling the diameter of the colonies can reduce the variation in spheroid diameter. In contrast, the ECM patterning method using a microstamp, shown in our previous study [34], did not provide perfect control of the ECM dot diameter. The microstamped ECM dots exhibited blurring, chipping, bleeding, and dirt on the base surface outside the dot (Figure 3c). Because the ECM adhering to the top surface of the stamp pillar was transferred to the substrate as a dot pattern, the dot pattern shape tended to change depending on the amount of ECM that the stamp could hold and the stamping pressure. Therefore, reproducibility was not sufficient. In addition, because an extremely small amount of ECM was retained on the top surface of the stamp pillar, the ECM tended to dry out before cell seeding. This could have been the cause of the reduced cell adhesion performance [51]. The ECM viscosity of a material is an important factor in the microstamping method. Highly viscous materials, such as Matrigel, are relatively easy to stamp, but a fragmented laminin-like iMatrix, which has a small molecular weight and low viscosity, is not easily transferred onto the fluoropolymer surface. However, considering the future applications of spheroids in drug discovery and regenerative medicine, it is desirable to use non-animal-derived materials, such as iMatrix, which is made from silk. Silk contains various functional proteins with antimicrobial properties. This strengthens functional silk research as it is a material with expected future medical applications. Examples include biofilms, hydrogels, sutures, culture scaffolds, and other biomaterials that utilize diverse silk fibers and silk proteins [52,53,54]. Therefore, our new method must be able to form iMatrix dots reliably.

These problems can be solved simultaneously with the new resin mask method. As is shown in Figure 3d, a uniform dot pattern can be obtained without failure, regardless of the number of repetitions. In this process, the ECM molecules floating in the diluent settle during incubation and adhere uniformly over the entire surface of the substrate, including the resin mask. A sufficient settling time ensured that a sufficient amount of ECM was retained in the dot holes. Although the amount of ECM was sufficient for cell adhesion, the dot layer was thin, resulting in a nearly flat 2D culture scaffold. Because the resin mask was peeled off in the liquid just before cell seeding, the ECM dots did not dry out. This technique is very convenient, because no additional time or effort is required to coat the ECM compared with the process used to coat ECM in a normal culture dish. In addition, unlike microstamping, the resin mask covers the uncoated surface, so there is no unintentional ECM adhesion to unintended areas and the pattern does not shift.

The resin masks were fabricated using photolithography, a technique commonly used in semiconductor fabrication. Lithography is a technique for fabricating microstructures by forming patterns in exposed and unexposed areas using photosensitive materials. The evolution of semiconductors has enabled remarkable progress in lithography technology. The resin used in this study is an adhesive intended for bonding medical materials, such as syringe needles. This material conformed to ISO 10993-5 standards (cytotoxicity). Because this material was not originally intended for lithography, it cannot be used for high-precision microfabrication, but we believe that its performance is sufficient for the target size of 2D cell colonies.

The spheroid dimensional controllability was demonstrated in Section 3.2, and the diameters of all the spheroids are represented in a histogram in Figure 4c–e. The standard deviations were very small in all cases: 9.0% at 300 μm, 10.1% at 400 μm, and 14.1% at 600 μm of the mean values. No spheroid production method has reported such a small variation in size. The superiority of this method can also be demonstrated in terms of dimensional control. We attribute these results to the fact that the number of cells constituting spheroids is governed by the area of the ECM dots. In our method, cells grow in a monolayer on the ECM dots. Therefore, the spheroid volume is proportional to the number of cells composing it and is determined by the area of the ECM dots. Upon accidental adhesion by collision, as in bioreactors, the speed can be controlled. However, the variation is difficult to control. In the case of low-adhesion plates with dimples, the number of cells in each dimple varies, even if the plates are seeded uniformly. Another factor may be that dead cells degraded via enzymatic treatment were incorporated into the spheroids. The high viability of trypan blue staining suggests that spheroids prepared by this method contain only a small number of dead cells. Therefore, the process of 2D culture with adhesion to the scaffold just before spheroidization and aggregation into spheroids without mechanical/chemical damage appears effective in maintaining high viability.

The localization of undifferentiated marker proteins (OCT3/4, SOX2, NONOG, SSEA4, TRA-1-60, and TRA-1-81) was confirmed the next day and on day 4 after seeding the hiPSCs. These results indicate that the method and substrate did not unexpectedly induce the differentiation of the hiPSC. Further extension of the culture period to 8 days showed that the expression of undifferentiated marker proteins decreased, and differentiation progressed. This may be attributed to the over-confluent state of the cells after 8 days of culture. This is a natural result because hiPSCs are usually passaged after approximately 3–5 days to prevent changes in cell characteristics due to stress caused by the over-confluent state. Therefore, in this method, it is important to control the over-confluent state in the cell culture stage.

In addition, the localization of differentiation marker proteins also confirmed that the colonies of dot pattern produced by this method are capable of differentiation. Unlike the conventional method, this method can produce spheroids without dissociating the cells and maintaining the cell polarity formed in 2D culture. Therefore, it is possible to easily and efficiently produce spheroids of uniform quality during differentiation induction.

## 5. Conclusions

We fabricated a resin mask with a UV-curing resin to form ECM dot patterns on fluoropolymer scaffolds. We cultured hiPSCs on ECM dot patterns formed using a new method. We confirmed the effectiveness of the spheroidization method in which cell colonies were detached from the substrate and cultured. In addition, we confirmed that spheroidization is possible without chemical (enzyme) treatment or physical stimulation, as in the microstamping method, and that a high size control performance could be achieved by improving the precision of the ECM dot fabrication using resin masks. We also confirmed that this method can maintain highly undifferentiated properties without adversely affecting the cells during the period required for inducing differentiation. Therefore, spheroids can be easily obtained simply by conducting normal cell culture using the fabricated substrate, and these spheroids represent a relatively simple and low-cost technology. Furthermore, we confirmed the existence of a differentiation potential by performing an initial induction of differentiation. In the future, we will evaluate further differentiation induction and establish a method for producing spheroids/organoids from size-controllable hiPSCs. Such spheroids/organoids are expected to be useful in drug discovery and regenerative medicine.

## Data Availability

Data supporting the findings of this study are available from the corresponding author upon request.
